# Voluntary exercise improves pulmonary inflammation through NF-κB and Nrf2 in type 2 diabetic male rats

**DOI:** 10.22038/IJBMS.2023.70416.15307

**Published:** 2024

**Authors:** Seyed Zanyar Athari, Fariba Mirzaei Bavil, Rana Keyhanmanesh, Hajie Lotfi, Yousef Sajed, Aref Delkhosh, Fariba Ghiasi

**Affiliations:** 1 Student Research Committee, Tabriz University of Medical Sciences, Tabriz, Iran; 2 Department of Physiology, Faculty of Medicine, Tabriz University of Medical Sciences, Tabriz, Iran; 3 Drug Applied Research Center, Tabriz University of Medical Sciences, Tabriz, Iran; 4 Tuberculosis and Lung Disease Research Center, Tabriz University of Medical Sciences, Tabriz, Iran; 5 Cellular and Molecular Research Center, Research Institute for Prevention of Non-Communicable Disease, Qazvin University of Medical Sciences, Qazvin, Iran; 6 Department of Pathobiology, Faculty of Veterinary Medicine, Division of Pathology, Urmia University, Urmia, Iran

**Keywords:** Diabetes mellitus, Inflammation, Lung, NF-κB, Nrf2, Voluntary exercise

## Abstract

**Objective(s):**

This study aimed to evaluate the effects of voluntary exercise as an anti-inflammatory intervention on the pulmonary levels of inflammatory cytokines in type 2 diabetic male rats.

**Materials and Methods:**

Twenty-eight male Wistar rats were divided into four groups (n=7), including control (Col), diabetic (Dia), voluntary exercise (Exe), and diabetic with voluntary exercise (Dia+Exe). Diabetes was induced by a high-fat diet (4 weeks) and intraperitoneal injection of streptozotocin (35 mg/kg), and animals did training on the running wheel for 10 weeks as voluntary exercise. Finally, the rats were euthanized and the lung tissues were sampled for the evaluation of the levels of pulmonary interleukin (IL)-10, IL-11, and TNF-α using ELISA, and the protein levels of Nrf-2 and NF-κB using western blotting and tissue histopathological analysis.

**Results:**

Diabetes reduced the IL-10, IL-11, and Nrf2 levels (*P*<0.001 to P<0.01) and increased the levels of TNF-α and NF-κB compared to the Col group (*P*<0.001). Lung tissue levels of IL-10, IL-11, and Nrf2 in the Dia+Exe group enhanced compared to the Dia group (*P*<0.001 to *P*<0.05), however; the TNF-α and NF-κB levels decreased (*P*<0.001). The level of pulmonary Nrf2 in the Dia+Exe group was lower than that of the Exe group while the NF-κB level increased (*P*<0.001). Moreover, diabetes caused histopathological changes in lung tissue which improved with exercise in the Dia+Exe group.

**Conclusion:**

These findings showed that voluntary exercise could improve diabetes-induced pulmonary complications by ameliorating inflammatory conditions.

## Introduction

Respiratory complications are some of the most important challenges in the significant proportion of patients with diabetes (1). Diabetic complications such as glycosylation of the chest muscles and bronchial wall proteins increased basal lamina thickness, hyperglycemia, inflammation, and oxidative stress can cause lung fibrosis, microangiopathy, impaired lung function, and respiratory problems (2). Several etiologies are involved in the development of type 2 diabetes including oxidative stress, macrophage-derived cytokines, T cells, and genetic and environmental factors, but an accurate understanding of the molecular mechanism still requires more studies (3). This systemic disease causes the destruction of blood vessels in many organs of the body such as kidneys, retina, and cardiovascular system (4). The lung as a tissue rich in alveolar and capillary networks may target capillary destruction in diabetic conditions. Molecular mechanisms that are more closely linked to diabetes and pulmonary disorders include pro-inflammatory pathways and vascular inflammation (1). In chronic conditions, gene encoding pro-inflammatory cytokines such as interleukin (IL)-1, IL-2, IL-6, Tumor necrosis factor (TNF)-α, and Monocyte chemoattractant protein (MCP)-1 play crucial roles in diabetes (5). Nuclear factor erythroid 2–related factor 2 (Nrf2) and Nuclear factor kappa-light-chain-enhancer of activated B cells (NF-κB) are key pathways regulating the delicate balance of cellular redox status and responses to stress and inflammation (6). The inhibition of NF-κB activation by phospholipid hydroperoxide glutathione peroxidase and 15-lipoxygenase is concomitant to the up-regulation of HO-1, probably via Nrf2 activation. On the other hand, the NF-κB p65 subunit represses the Nrf2-anti-oxidant responsive element (ARE) pathway at the transcriptional level (7). It has been reported that Nrf2 deficiency enhanced NF-κB-mediated pro-inflammatory reactions and increased the pro-inflammatory genes which regulate NF-κB, such as the interleukins and TNF-α. Moreover, NF-κB suppressed the transcriptional activity of Nrf2 (8).

Some of the most common pharmacological agents used to treat the complications of type 2 diabetes include drugs from different classes such as biguanides, sulfonylureas, meglitinides, thiazolidinediones, and insulin (9). Long-term usage of these drugs for diabetes management has many side effects and complications, leading to cardiovascular disorder, disturbances of the liver and kidney, and weight gain. Also, these drugs can interfere with other non-diabetic medications for a long time (9). Therefore, using non-pharmacological treatment such as exercise can be an effective method in the management of the disease.

According to previous studies, exercise has anti-inflammatory effects, and regular exercise for an extended period can prevent chronic diseases (10). Exercise can activate the expression of cellular anti-oxidant systems, and there is evidence to suggest that Nrf2 plays a critical role in this regard (11). A study demonstrated that exercise could improve inflammation and reduce oxidative stress by inhibiting NF-κB and activating Nrf2 signaling pathways in the lung tissue (12). In human diabetic patients, it has been reported that regular exercise caused an increase in systemic anti-inflammatory cytokines such as IL-4 and IL-10 and a decrease in the levels of C-Reactive Protein (CRP), IL-6, and TNF-α (11). It has been shown that regular physical activity can decrease the risk of diabetes mellitus and prevent the progression of this disease (13). Considering the side effects of common drugs in treating diabetes and the beneficial effects of exercise in the treatment of type 2 diabetes mellitus, this study aimed to investigate the effects of voluntary exercise on the inflammatory cytokines of lung tissue, NF-κB, and Nrf2 in type 2 diabetic male rats.

## Materials and Methods

***Ethics***

All study procedures and interventions related to animal behavior have been performed based on the principles and ethical considerations approved by Tabriz University of Medical Sciences (Ethical number: IR.TBZMED.VCR.REC.1399.241).

***Animals***

In this study, 28 male Wistar rats weighing 200±20 gr that were purchased from the Pasteur Institute (Tehran, Iran) were used. The animals were kept in standard conditions (22±2 °C, 12/12 hr dark-light cycle) and had free access to water and food. One week after adaptation to the laboratory environment, the animals were randomly divided into four groups (each n=7); including the Control group (Col), Diabetic group (Dia), Voluntary Exercise group (Exe), and Diabetic and voluntary exercise group (Dia+Exe). No intervention was performed in the Col group, animals received only citrate buffer (streptozotocin solvent). The animals of the Dia group became diabetic and were kept for ten weeks. In the Exe group, animals performed voluntary exercise using the running wheel for ten weeks. The animals of the Dia+Exe group became diabetic and did voluntary exercise using a running wheel for ten weeks after confirming diabetes.

***Diabetes induction***

For induction of type 2 diabetes, the high-fat diet (HFD) followed by low-dose streptozotocin (STZ) injection (35 mg/kg, intraperitoneally) is used to induce insulin resistance and develop mild dysfunction in β-cells without complete elimination of insulin secretion. This model mimics the development of type 2 diabetes in humans (14). After 5 days of STZ injection, animals with blood glucose higher than 250 mg/dl were reflected as diabetic and selected for further studies.

***Voluntary exercise***

The voluntary exercise was performed for ten weeks by placing the animal in a special cage with a rotating wheel, and animals that had less than 2,000 rotations in 24 hr were excluded from the study. This voluntary exercise was acknowledged as a mild-to-moderate exercise (15).

***Tissue sampling***

At the end of the experiment, intraperitoneally injections of ketamine (90 mg/Kg) and xylazine (10 mg/Kg) were used for anesthesia then they were euthanized by using a guillotine, and lung tissues were sampled. The lungs were then washed out with cold, sterile normal saline and immediately frozen in liquid nitrogen. All samples were stored in a -80 °C freezer until parameter analysis.

***Western blotting***

For protein extraction, after homogenizing a little amount of the upper part of the frozen lung in ice-cold Radioimmunoprecipitation assay buffer (RIPA), lysis buffer was added and the mixture was centrifuged at 14,000 rpm for 10 min at 4 °C. Supernatants were obtained, stored at −80 °C, and finally, the protein levels were determined using Bradford’s technique. The cell lysates (50 µg protein/lane), isolated via sodium dodecyl sulfate-polyacrylamide gel electrophoresis (SDS-PAGE), were loaded and transferred on the polyvinylidene difluoride (PVDF) membranes (Millipore, Billerica, MA, USA). The membranes were incubated via primitive antibodies overnight at 4 °C with goat anti-rabbit IgG (H+L) Cross-Adsorbed secondary antibody and Alexa Fluor 594 (R37117), and immersed in ECL Plus Western Blotting detection reagent and displayed on Hyper film ECL (both from Amersham, Piscataway, NJ, USA). The Lab Works 4.5 software (UVP, Upland, CA, USA) was used for calculating the band’s intensity. NF-κB (sc-74465) (Santa Cruz Biotechnology, Inc.), Nrf2 (Lys382), and β-actin (sc-47778) primary antibodies (Cell Signaling Technology, #2525) were used for western blotting (16).

***Elisa***

Lung tissue samples were homogenized in 10 volumes of 50 mM sodium phosphate buffer (pH 7.4) at 4 °C and centrifuged at 4.500×g for 15 min, and the supernatants were collected for testing IL-10, IL-11, and TNF-α concentrations. Commercially available rat enzyme-linked immune sorbent assay (ELISA) kits (Bioassay Technology Laboratory, Shanghai Kora in Biotech Co., China) was used to determine the tissue levels of IL-10, IL-11, and TNF-α concentrations according to the instructions of the manufacturers (17).

***Histopathology***

The left diaphragmatic lobe of the lung, fixed in 10% buffered formalin and embedded in paraffin, is used to evaluate histopathological changes. The tissue sections (5-µm thick) were stained with hematoxylin and eosin (H&E) and examined by experienced pathologists using an Olympus light microscope (Olympus, Tokyo, Japan). All measurements and scoring were performed on blinded slides. The tissue changes including the peribronchiolar infiltration of inflammatory cells, alveolar septal thickness, vascular hyperemia, and emphysema were semi-quantified as follows: 0; normal, 1+; mild change, 2+; moderate change, and 3+; severe change.

***Data analysis***

All results of the present study were reported as mean± Standard Error of the Mean (SEM). SPSS 16 statistical software was used for statistical analysis. The data normality was examined by the Kolmogorov-Smirnov test, and one-way analysis of variance (ANOVA) followed by LSD *post-hoc* was performed to analyze the factors in lung tissue samples. To analyze histological changes between different groups, the Kruskal-Wallis test was used. The diagrams were drawn using Graph pad Prism 8 software. In all cases, *P*<0.05 was considered statistically significant.

## Results

***Effect of voluntary exercise on the levels of IL-10, IL-11, and TNF-α in lung tissue of diabetic rats***

The results of this study demonstrated that induction of diabetes significantly decreased pulmonary levels of IL-10 and IL-11 in the Dia group compared to the Col group (*P*<0.01). The voluntary exercise increased the level of this cytokine in the lung tissue of the Exe and Dia+Exe groups compared to the Dia group (*P*<0.01 to *P*<0.05). There are no significant differences between IL-10 and IL-11 of Exe and Dia+Exe groups as well as those of Exe and Col groups (Figure 2A and 2B). The level of IL-11 was significantly elevated in Exe and Dia+Exe groups compared to the Dia group (*P*<0.001 to *P*<0.05, Figure 2B). 

There was a significant increment in the pulmonary level of TNF-α in the Dia group compared to controls (*P*<0.001). This parameter was significantly decreased in Exe and Dia+Exe groups compared to the Dia group (*P*<0.001). The levels of TNF-α in the Col and Exe groups were not significantly different (Figure 2C).

***Effect of voluntary exercise on the expression of NF-kB in lung tissue of diabetic rats***

Western blot analysis demonstrated a significant increase in the level of NF-kB in lung tissue of the Dia group compared to the Col group (*P*<0.001). This factor was significantly decreased in the Exe (*P*<0.001) and Dia+Exe (*P*<0.001) groups compared to the Dia group. NF-kB level in the Dia+Exe group was significantly higher than those of the Col (*P*<0.01) and Exe (*P*<0.001) groups (Figure 3).

***Effect of voluntary exercise on the expression of Nrf2 in lung tissue of diabetic rats***

The results of western blot analysis showed a significant decrease in lung tissue level of Nrf2 in the Dia group compared to the Col group (*P*<0.001). However, the pulmonary level of Nrf2 in the Exe group was significantly higher than that of the controls (*P*<0.001). This parameter was significantly increased in the Exe and Dia+Exe groups compared to the Dia group (*P*<0.001). The level of Nrf2 in the lung tissue of the Dia+Exe group was significantly lower than that of the Exe group (*P*<0.001, Figure 4).

***Effect of voluntary exercise on the histopathological changes in lung tissue of diabetic rats***

The results of histopathological examination revealed no significant changes in the lung tissue of Col (I) and Exe (III) groups, however, a mild degree of emphysema was seen in the Exe group (point mark, III). The peribronchiolar infiltration of inflammatory cells (upper point mark, II), increased alveolar septal thickness, and vascular hyperemia (lower point mark, II) have been seen in the Dia group (II). In Dia + Exe group (IV), the peribronchiolar infiltration of the inflammatory cells was reduced, and the rate of hyperemia was lower than that of the Dia group (II) (Figures 5 and 6).

## Discussion

One of the most important issues in the pathophysiology of diabetes is inflammation (18). Chronic hyperglycemia which is one of the hallmarks of diabetes can lead to glycosylated proteins with pro-inflammatory effects and complications in small vessels (19). Studies have demonstrated that diabetes can induce pulmonary mononuclear cell invasion, cell proliferation, interstitial cell hypertrophy, and increased pulmonary vascular permeability and fibrosis followed by interstitial enlargement, which eventually leads to the collapse of the alveolar space (20–22). Recent studies have shown that lung volume is inversely related to the level of systemic inflammation (23, 24). On the other hand, diabetes is associated with increased inflammatory mediators such as C-reactive protein (CRP) and interleukin (IL)-6 (25). Systemic and localized inflammation in type 2 diabetes may affect the lungs and other organs, leading to several complications such as decreased lung function, metabolic syndrome, and atherosclerosis (19). As seen in our study, pro-inflammatory factors increased and anti-inflammatory factors decreased following the induction of diabetes. The gene encoding pro-inflammatory cytokines such as IL-1, IL-2, IL-6, Tumor necrosis factor (TNF)-α, and Monocyte chemoattractant protein (MCP)-1 play significant roles in increasing the risk of chronic diseases such as asthma, atherosclerosis, and rheumatoid arthritis (5, 26). These genes are regulated by nuclear factor-κB (NF-κB), a transcription factor in the expression of pro-inflammatory proteins (27).

Metabolic diseases such as diabetes are associated with reduced IL-10, an anti-inflammatory cytokine produced by macrophages and lymphocytes (28). IL-10 exhibits its anti-inflammatory activity by inhibiting phosphorylation of nuclear factor kappa B (I*κ*B) kinase (29). Zhu *et al*. stated that IL-10 activates AMP-activated protein kinase (AMPK), and AMPKα1 is essential for its anti-inflammatory function (30). High glucose levels reduce AMPK activity which has a key role in inflammation, hence type 2 diabetes mellitus-induced inflammation interferes with IL-10 signaling (31). It has been concluded that high glucose levels in patients with type 2 diabetes could inhibit the suppression of TNF-α by IL-10 because of its disrupted messaging pathway which can cause insulin resistance (32). In line with our study, previous research has shown that exercise not only reduced TNF-α levels but also increased IL-10 levels in normal and inflammatory conditions (33, 34).

IL-11 is another chronic inflammatory biomarker in patients with type 2 diabetes. This cytokine has an inhibitory role in producing pro-inflammatory cytokines (IL-6, IL-1β, and TNF-α) (35). As revealed in the present study, diabetes induction diminished IL-11 in lung tissue, and exercise could increase its pulmonary level. Lgssiar *et al*. have stated that IL-11 could prevent diabetes and improve inflammatory status (36). It has been suggested that voluntary exercise might be effective to inhibit the NF-κB pathway in lung tissue (37). Researchers surveyed the pulmonary expression of NF-κB and TNF-α genes in the lung tissue of rats after four weeks of aerobic exercise has decreased, and aerobic exercise has been shown to exert anti-inflammatory effects in the lung tissue (38).

NF-κB is momentous in type 2 diabetes; it can regulate some cytokines in the development of insulin resistance, such as TNF-α, IL-1, and IL-6 (39). In the present study, the pulmonary NF- κB level was increased after induction of diabetes. NF-κB is activated by various factors such as cytokines, reactive oxygen species (ROS) (40), high sugar (41), free fatty acids (42), and IκB. Beta and alpha IκB isoforms inhibit NF-κB activity. When NF-κB dissociates from the inhibitory protein, it is transported into the nucleus and activates the transcription of inflammatory genes such as IL-1 and IL-6 (43). TNF-α is a potent activator of NF-κB, which induces insulin resistance through serine phosphorylation of the insulin receptor substrate-1 (IRS1) (44). Previous studies have shown that TNF-α which is stored in the adipose tissue of obese animals and humans can lead to insulin resistance in obese individuals (44, 45). Therefore, one of the pathological reasons for insulin resistance in diabetic cases is elevation of TNF-α, which was also observed in the present investigation.

Nuclear factor erythroid 2–related factor 2 (Nrf2) is one of the factors involved in the anti-oxidant signaling pathway which regulates anti-oxidant defense gene expression levels (46, 47). The expression of this factor is high in the kidney, liver, heart, and lungs (48). In addition to the mentioned role of NF-κB factor in causing inflammation in diabetes, its interaction with the Nrf2 factor has also been reported (49). It has been reported that Nrf2 in pancreatic beta cells in diabetes can suppress inflammation by modulating inflammation and the major systems of cellular protein degradation, namely proteasome and autophagy in pancreatic beta cells (50, 51). 

Diabetes mellitus can lead to oxidative stress conditions that result in elevated ROS levels and decreased anti-oxidant defenses (52), the Nrf2-Keap1 (Kelch-like ECH-associated protein1) signaling pathway is one of the best-known of these molecular mechanisms (53). According to the findings of the present study, induction of diabetes decreased the amount of Nrf2 levels and exercise could reverse this situation. Nrf2 can play a protective role in response to oxidative stress (54) and increase insulin sensitivity, regulate glucose metabolism, mitochondrial bioenergy, and lipid metabolism, as well as reducing inflammation and improving drug metabolism (55). Mallard *et al.* in 2020 revealed that exercise could increase almost all molecular aspects of the Nrf2-ARE (anti-oxidant response element) pathway (56). In line with our study, Tsou and his colleagues showed that exercise could increase the anti-oxidant capacity in dopaminergic neurons by activating the Nrf2 pathway (57). After oxidative stress exposure, Nrf2 dissociated from inactivated Keap1 (Nrf2 inhibitor) and after phosphorylation, transferred to the nucleus and bound to anti-oxidant responsive element (ARE) sites, which results in the expression of downstream genes such as glutathione peroxidases and heme oxygenase-1 (58). Decreased Nrf2-Keap1 signaling pathway seems to have a close association with diabetes mellitus complications such as diabetic retinopathy and cardiomyopathy. Activation of the Keap1-Nrf2 system could reduce the damage induced by oxidative stress and inflammation in diabetes (59). 

**Figure 1:**
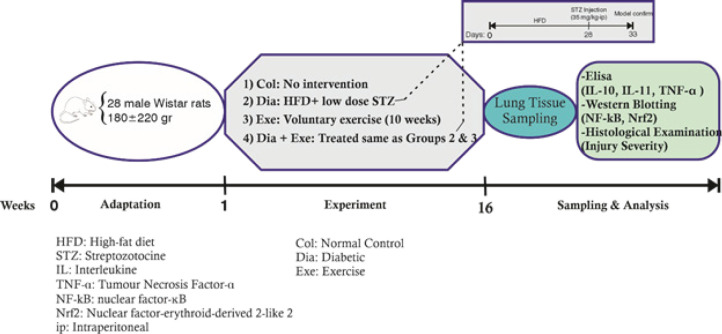
Timeline of this investigation

**Figure 2:**
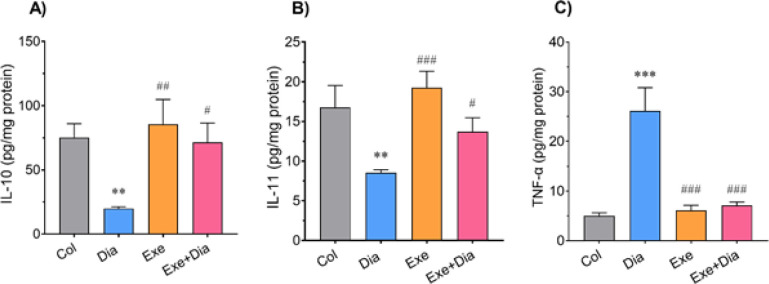
Effect of exercise on the levels of A) IL-10, B) IL-11, and C) TNF-α in the lung tissue of type 2 diabetic male rats

Data are expressed as mean±SEM (n=7). Statistical differences among the groups were evaluated by one-way analysis of variance (ANOVA), followed by an LSD *post-hoc* test.

** *P*<0.01 vs Col group. #*P*<0.05; ##*P*<0.01; ###*P*<0.001 vs Dia group.

Col: Control; Dia: Diabetic; Exe: Exercise; Dia+Exe: Diabetic+Exercise; IL: Interleukin; TNF-α: Tumor necrosis factor-α

**Figure 3:**
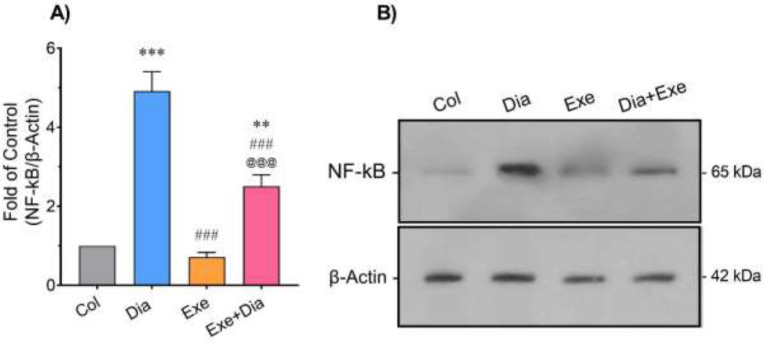
A) Quantitative densitometric analysis of the protein level of NF-kB in the lung tissue of different groups. B) Immunoblotting images of the expression of NF-kB and β-Actin in the lung tissue of type 2 diabetic male rats

Data are expressed as mean±SEM (n=7). Statistical differences among the groups were evaluated by one-way analysis of variance (ANOVA), followed by an LSD *post-hoc *test.

**P<0.01; ***P<0.001 vs Col group. ###*P*<0.001 vs Dia group. @@@*P*<0.001 vs Exe group.

Col: Control; Dia: Diabetic; Exe: Exercise; Dia+Exe: Diabetic+Exercise

**Figure 4:**
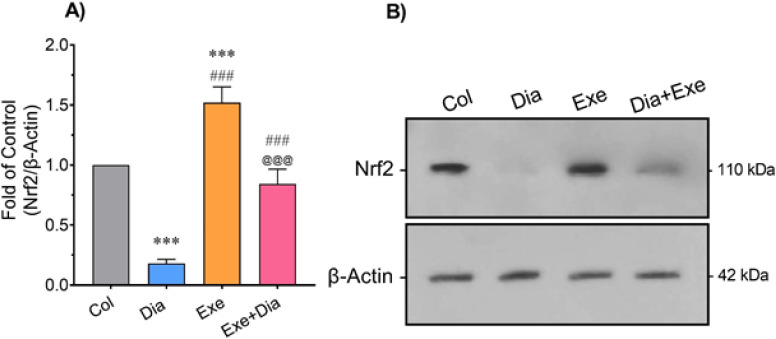
A) Quantitative densitometric analysis of Nrf2 in different groups. B) Immunoblotting images of the expression of Nrf2 and β-Actin in the lung tissue of type 2 diabetic male rats

Data are expressed as mean±SEM (n=7). Statistical differences among the groups were evaluated by one-way analysis of variance (ANOVA), followed by an LSD *post-hoc* test.

***P<0.001 vs Col group. ###*P*<0.001 vs Dia group. @@@*P*<0.001 vs Exe group.

Col: Control; Dia: Diabetic; Exe: Exercise; Dia+Exe: Diabetic+Exercise

**Figure 5:**
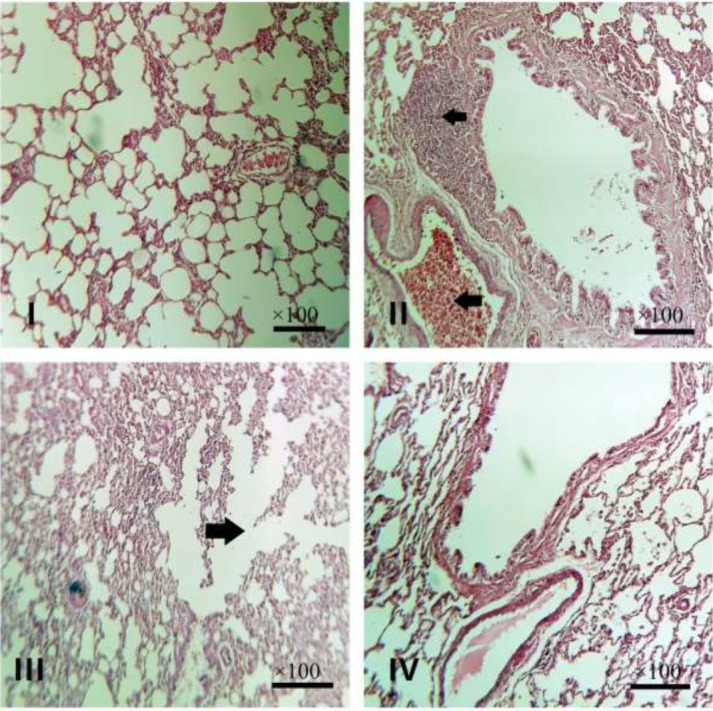
Microscopic view of the lung tissues of type 2 diabetic male rats, the peribronchiolar infiltration of inflammatory cells (upper point mark, II), increased alveolar septal thickness, vascular hyperemia (lower point mark, II), and a mild degree of emphysema was seen (point mark, III). (hematoxylin & eosin staining, ×100)

The point marks show the changes. Statistical differences among the groups were evaluated by one-way analysis of variance (ANOVA), followed by an LSD *post-hoc* test.

Col group (I), Dia group (II), Exe group (III), Dia + Exe group (IV)

**Figure 6:**
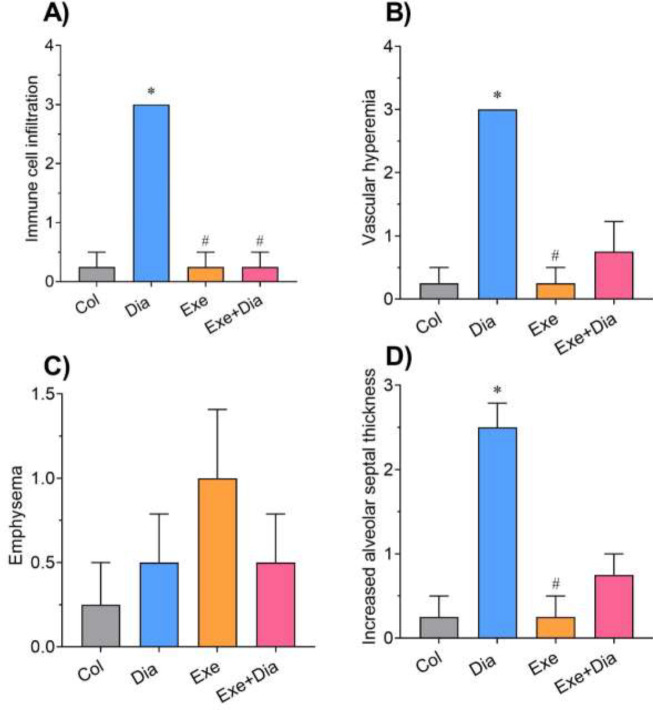
Effect of Exercise on A) infiltration of inflammatory cells, B) vascular hyperemia, C) emphysema, and D) increased alveolar septal thickness of type 2 diabetic male rats

Data are expressed as mean±SEM (n=7).

Statistical differences among the groups were evaluated by the Kruskal-Wallis test.

* *P*<0.05 vs Col group. #*P*<0.05 vs Dia group.

Col: Control; Dia: Diabetic; Exe: Exercise; Dia+Exe: Diabetic+Exercise

## Conclusion

The present findings showed that voluntary exercise could have therapeutic effects on diabetes mellitus type 2, and it improved its pulmonary complication by elevation of the levels of Nrf2, IL-10, and IL-11 as well as decreasing TNF-α and NF-kB levels. In histopathological results, the peribronchial inflammatory cell infiltration and the rate of hyperemia were improved concomitant with biochemical findings.

## Authors’ Contributions

SZ A helped with data curation, formal analysis, and writing the original draft. F MB provided supervision, validation, and visualization. R K helped validate, visualize, write, review, and edit. H L and Y S performed data curation. A D studied the histopathological changes. F G contributed through conceptualization, data curation, formal analysis, methodology, project administration, supervision, validation, visualization, writing the original draft, and reviewing and editing.

## Funding

F G has received research support from the Tuberculosis and Lung Disease Research Center, Tabriz University of Medical Sciences, Tabriz, Iran (grant number: 64651).

## Data Availability Statement

The data that support the findings of this study are available from the corresponding author upon reasonable request.

## Etical Declaration

All study procedures and interventions related to animal behavior have been performed based on the principles and ethical considerations of working with laboratory animals approved by Tabriz University of Medical Sciences, Iran (Ethical number: IR.TBZMED.VCR.REC.1399.241).

## Conflicts of Interest

No potential conflicts of interest were reported by the authors.
